# Silicon photon-counting detector for full-field CT using an ASIC with adjustable shaping time

**DOI:** 10.1117/1.JMI.7.5.053503

**Published:** 2020-10-06

**Authors:** Christel Sundberg, Mats Persson, Martin Sjölin, J. Jacob Wikner, Mats Danielsson

**Affiliations:** a KTH Royal Institute of Technology, Physics of Medical Imaging, Stockholm, Sweden; b Linköping University, Department of Electrical Engineering, Linköping, Sweden

**Keywords:** silicon photon-counting detector, dose efficiency, photon-counting detector, shaping time, application-specific integrated circuit

## Abstract

**Purpose:** Photon-counting silicon strip detectors are attracting interest for use in next-generation CT scanners. For CT detectors in a clinical environment, it is desirable to have a low power consumption. However, decreasing the power consumption leads to higher noise. This is particularly detrimental for silicon detectors, which require a low noise floor to obtain a good dose efficiency. The increase in noise can be mitigated using a longer shaping time in the readout electronics. This also results in longer pulses, which requires an increased deadtime, thereby degrading the count-rate performance. However, as the photon flux varies greatly during a typical CT scan, not all projection lines require a high count-rate capability. We propose adjusting the shaping time to counteract the increased noise that results from decreasing the power consumption.

**Approach:** To show the potential of increasing the shaping time to decrease the noise level, synchrotron measurements were performed using a detector prototype with two shaping time settings. From the measurements, a simulation model was developed and used to predict the performance of a future channel design.

**Results:** Based on the synchrotron measurements, we show that increasing the shaping time from 28.1 to 39.4 ns decreases the noise and increases the signal-to-noise ratio with 6.5% at low count rates. With the developed simulation model, we predict that a 50% decrease in power can be attained in a proposed future detector design by increasing the shaping time with a factor of 1.875.

**Conclusion:** Our results show that the shaping time can be an important tool to adapt the pulse length and noise level to the photon flux and thereby optimize the dose efficiency of photon-counting silicon detectors.

## 1 Introduction

Over the past years, photon-counting spectral detectors for computed tomography (CT) have received considerable attention. Compared to conventional energy-integrating CT detectors, in which the energy of many photons is integrated over a certain time period, photon-counting spectral detectors count the individual photons and measure their energies. This enables higher signal-to-noise ratio (SNR), higher spatial resolution, and improved spectral imaging.^1^^–^^3^

Although most research into photon-counting spectral CT detectors is centered around CdTe (cadmium telluride) and CZT (cadmium zinc telluride),^4^^,^^5^ silicon is also a candidate detector material.^6^^,^^7^ An advantage of silicon compared to CdTe and CZT is that it is possible to manufacture as almost perfect crystals with fast charge collection that is robust even at very high count rates. The low atomic number results in no cross-talk due to K-fluorescence, but on the other hand, there is a significant fraction of Compton interactions. In Compton interactions, only a part of the photon energy is deposited. This results in counts with little to no energy information. However, as secondary interactions can be largely avoided using tungsten shielding, the registered Compton counts still correspond to unique photons. For density imaging tasks, these counts are therefore vital and contribute to both dose efficiency and contrast in the image. In spectral imaging, we have previously shown that the contrast can be increased with 5% to 14% by including Compton counts.^6^

**Fig. 1:**
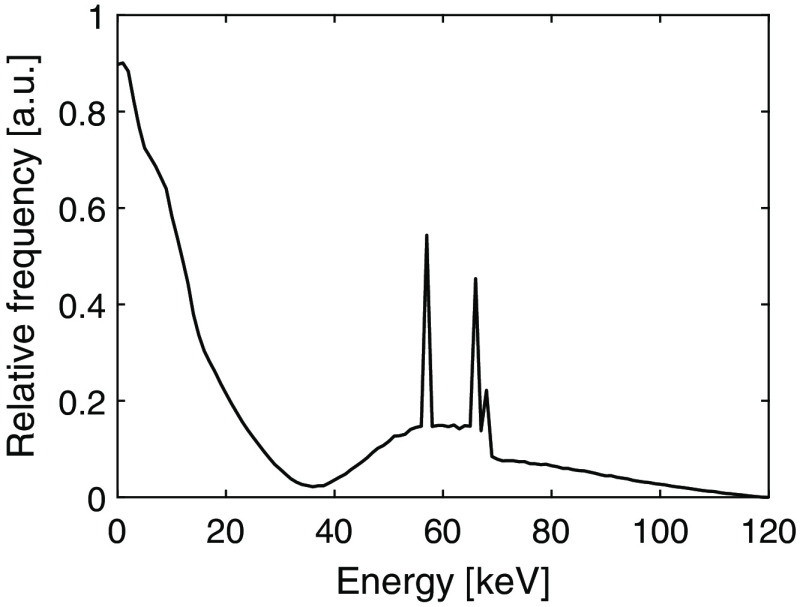
Simulated spectrum of the deposited energies in a silicon detector from an x-ray source operated at 120 kVp, including 20 cm soft tissue filtration between the source and the detector.

Figure 1 shows a typical spectrum of the deposited energies in a silicon detector. Compton interactions are visible as the peak between 0 and 35 keV and to obtain a high dose efficiency, it is desirable to have the lowest threshold as close to 0 keV as possible. However, if the threshold is set too low, electronic noise will give unwanted false counts. Having a low electronic noise is therefore vital for a high dose efficiency.

The electronic noise level is affected by the filter component in the readout electronics. While the shaping time remains shorter than microseconds, a longer shaping time leads to decreased noise, but this comes at the expense of increased pulse length. If two photons are registered within a time frame shorter than the pulse length they will add up, resulting in pileup. This can cause count loss or spectral distortion. Increasing the pulse length therefore reduces the ability of the detector to measure at high count rates.

For silicon detectors count-rate performance is not a problem.^8^^–^^10^ The relatively long absorption length of silicon compared to other detector materials enables segmentation in the direction of the incident x-rays. Each segment then consists of a row of readout channels that measure only a fraction of the incident x-ray flux. As the total number of absorbed photons becomes divided among the segments the demand on high count-rate capability is reduced in each readout channel. In situations where power consumption is not an issue, the optimal number of depth segments would ideally be chosen to minimize pileup without generating excessive charge sharing between the depth segments. We have previously presented systems with 9 and 16 depth segments that have shown good performance for diagnostically relevant count rates with linearities of detected count rates up to 90 and $200\;\mathrm{Mcps}/{\mathrm{mm}}^{2}$, respectively.^8^^–^^10^

For CT detectors in a clinical environment, power consumption is one of the largest system constraints and active cooling has to be employed. There is no hard limit for power consumption for future photon-counting detectors (including silicon based) but it is expected to be in the range of today’s CT detectors to avoid expensive installations. Depending on the detector width this typically means <5  kW. To decrease the power consumption, it is possible to decrease the number of depth segments since the power consumption scales with the number of readout channels. Decreasing the number of depth segments reduces the count-rate capability of the system and makes it more prone to pileup. To counteract pileup, it is desirable to have fast readout electronics with a short shaping time. If the power in each channel is kept constant, decreasing the shaping time leads to shorter pulses but also increased noise.

In a typical CT scan, the photon flux varies greatly between different projection lines. The number of electronic noise counts is largely unaffected by the photon flux, and at high count rates the electronic noise counts only constitute a small fraction of the total counts meaning that the impact of electronic noise counts on image quality is limited. At low photon fluxes, on the other hand, the false counts from electronic noise are more detrimental as they constitute a larger fraction of the total number of counts. However, at low fluxes there is no demand for short pulses, as there is no problem with pileup. In a detector with few depth segments, it would therefore be desirable to measure different projections with different shaping times, e.g., a long shaping time at low photon fluxes to reduce the electronic noise and potentially decrease the lowest threshold and a short shaping time to mitigate pileup at high photon fluxes.

Photon-counting detectors with multiple shaping times are commonly used in positron emission tomography and single photon emission computed tomography systems. In these systems, two shaping filters are used in parallel: one filter is equipped with a long shaping time and is used for energy classification, while the other filter has a short shaping time, which enables extraction of timing information.^11^^,^^12^ Detectors with dual shaping times have also been presented for correction of charge loss.^13^^,^^14^ In such systems, a short shaping time is used to measure the position of the event while the filter with the long shaping time provides energy information.

An alternative to decreasing the number of depth segments is to decrease the power consumption in each readout channel by reducing the power to the preamplifier. The preamplifier integrates the induced currents that result from photon interactions and is one of the main contributors of noise in the readout channel.^15^ The noise is dependent on the power at which the preamplifier is operated: as the power is decreased, noise increases. Similar to the case with fewer depth segments, it is therefore desirable to adapt the shaping time to the incident flux. Increasing the shaping time can then mitigate the noise increase that comes with decreasing the preamplifier power. For both of the presented alternatives to reduce the power consumption, the trade-off between pileup with long shaping times and high noise with short shaping times must also be taken into consideration.

In order to develop a detector with low power consumption, we propose using an adjustable shaping time that can be adapted according to the incident x-ray flux and thereby mitigate the trade-off between dose efficiency and count-rate capability as previously shown in a preliminary study.^16^ To experimentally demonstrate the potential of varying the shaping time, we present noise and pulse shape measurements from a prototype detector for two shaping times. Furthermore, we develop a simulation model of the detector system, which we fit to the measured pulse shapes, and we compare the measured noise level to the predictions of the model. We also propose a potential future detector channel design that is specifically adapted to use with an adjustable shaping time and investigate the potential for reducing the power consumption with this channel design.

## 2 Method

### 2.1 Readout Electronics

The detector readout is performed by a custom application-specific integrated circuit (ASIC). The ASIC is implemented in a 180-nm CMOS process and is based on an architecture previously presented by Gustavsson et al.^17^ that has been measured and evaluated for photon-counting spectral CT by Xu et al.^10^^,^^18^ It is a mixed-signal circuit capable of photon-counting and energy classification.

**Fig. 2:**
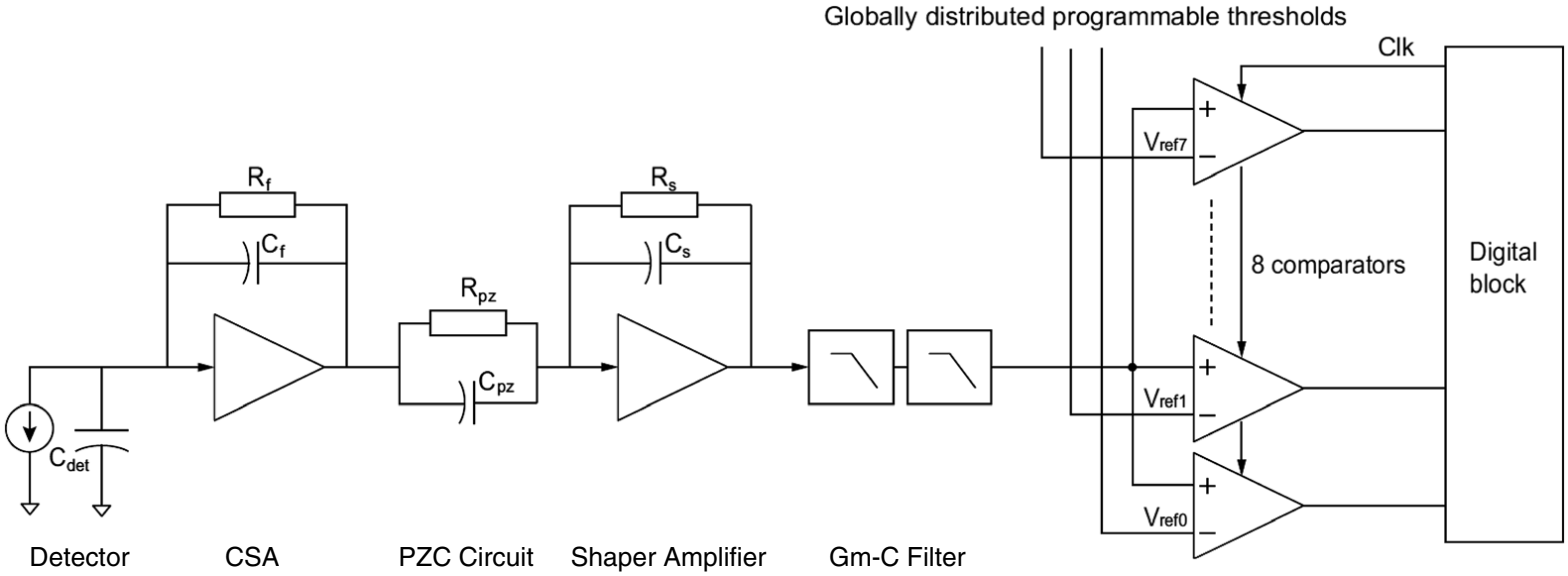
Block diagram of the first stages of the readout electronics, i.e., the interface to the detector diode. The CSA integrates the charge, the shaper amplifier, PZC circuit, and $G_{m}\text{-}C$ filter shape the pulse. The comparators discretize the pulse.

The ASIC has 160 channels in total and eight energy bins per channel. In Fig. 2, an overview of the analog interfacing circuits in each channel is presented. The first component is the preamplifier, which is a charge-sense amplifier (CSA) that integrates the current generated by incident photons and creates an output voltage. The CSA is followed by a pole zero cancellation (PZC) circuit and a shaper amplifier. A shaping filter is implemented after the shaper amplifier using two cascaded lossy transconductance-C ($G_{m}\text{-}C$) integrator stages. The two stages effectively form one pole each and combined act as a second-order filter with a shaping time τ that is set by the values of the transconductance $G_{m}$ and the capacitance C. For a hypothetical, ideal implementation of this circuit, the filter output, $V_{f}(s)$, can be expressed as

$$V_{f}(s)=\frac{1}{C_{f}}\frac{C_{\mathrm{pz}}}{C_{s}}\frac{\tau_{s}}{1+s\tau_{s}}{\left(\frac{2}{1+s\tau_{0}}\right)}^{2}I_{\text{in}}(s)$$ (1)

in the frequency domain, with $I_{\text{in}}(s)$ as the input from the detector. $C_{f}$ is the feedback capacitance of the CSA, $C_{\mathrm{pz}}$ is the capacitance of the PZC circuit, $C_{s}$ is the shaper amplifier capacitance, and s=jω where j is the imaginary unit and ω is the angular frequency. The expression also contains two time constants: $\tau_{s}$, which is the time constant of the shaper amplifier, and $\tau_{0}=\frac{\tau }{2}$, which is the filter time constant. The time constant $\tau_{s}$ is typically set to be in the order of the time constant of the filter: $\tau_{s}\approx \tau_{0}$.

In our current ASIC prototype, there is a parasitic load from the comparators at the filter output. We expect this to result in a time constant of the second $G_{m}\text{-}C$ stage that is twice as large as the time constant of the first stage. Furthermore, the current ASIC prototype supports two shaping time settings that are achieved by altering the capacitor values in the $G_{m}\text{-}C$ stages. The shaping time settings correspond to a long shaping time, which we expect to be ∼40  ns, and a short shaping time, which is dependent on the bandwidth of the amplifiers as well as parasitics varying with ASIC samples but expected to be in the range between 20 and 40 ns.

A general representation of the filter output from the ASIC, both prototype and ideal, is given as

$$V_{f}(s)=\frac{1}{C_{f}}\frac{C_{\mathrm{pz}}}{C_{s}}\frac{\tau_{s}}{1+s\tau_{s}}\frac{2}{\left(1+s\tau_{1}\right)}\frac{2}{\left(1+s\tau_{2}\right)}I_{\mathrm{in}}(s),$$ (2)

in which the time constant of the shaper amplifier, $\tau_{s}$, and the time constants of the two $G_{m}\text{-}C$ stages, $\tau_{1}$ and $\tau_{2}$, are defined by Table 1 for each ASIC type. In the prototype ASIC, we represent the impact of the shaping time setting on the time constants of the $G_{m}\text{-}C$ stages with a parameter B. There are two selectable values of B corresponding to the two shaping time settings: $B_{\text{long}}=1$ and $B_{\text{short}}$. With the shaping time defined as $\tau =2B\tau_{0}$, the two shaping time settings result in $\tau_{\text{long}}=2\tau_{0}$ and $\tau_{\text{short}}=2B_{\text{short}}\tau_{0}$. The value of $B_{\text{short}}$ is not known due to the above-mentioned comment but is assumed to be in the interval $0.5\leq B_{\text{short}}<1$ in accordance with the expected shaping time range of 20 to 40 ns. In the current ASIC prototype, we also expect the time constant $\tau_{s}$ to be nominally 14 ns.

**Table 1:** Time constants in the current ASIC prototype and the ideal ASIC.

	$\tau_{s}$	$\tau_{1}$	$\tau_{2}$
Current ASIC prototype	14 ns	$B\tau_{0}$	$2B\tau_{0}$
Ideal ASIC	$\tau_{0}$	$\tau_{0}$	$\tau_{0}$

In the time domain, $v_{f}(t)$ is the filter output voltage and each voltage peak corresponds to the energy deposited by a photon. To register the peak voltage of each pulse, the filter output is digitized by eight comparators. The voltage $v_{f}(t)$ is discretized into eight different levels using a set of controllable thresholds (energy bins), one in each comparator. The comparator outputs are sampled at a fixed time period, the clock cycle $\tau_{c}$, and whenever the sampled voltage exceeds the lowest set comparator threshold, a count is registered. The voltage pulses are typically longer than the clock cycle and each count is therefore followed by a deadtime to avoid multiple counts from a single photon event. The deadtime is a multiple of the clock cycle during which the height of the pulse is determined and the channel is unable to register new counts. The pulse is registered in the energy bin of the highest comparator threshold that has been triggered by the pulse.

#### 2.1.1 Shaping time

The pulse shaping is semi-Gaussian, and it is realized by lowpass filters with cut-off frequency determined by the shaping time. For shaping times that are shorter than a few microseconds, an increase of the shaping time results in a lower cut-off frequency.^15^ In the case of white noise, a lower cut-off frequency decreases the total noise power, but on the other hand, the cut-off frequency also affects the pulse height of the photons and thereby the gain in mV/keV. The SNR is here defined as the ratio between the gain and the standard deviation of the noise, σ. Therefore, in order for the SNR to increase when the shaping time is increased, the reduction of noise must be greater than the reduction of gain.

### 2.2 Measurement Setup

**Fig. 3:**
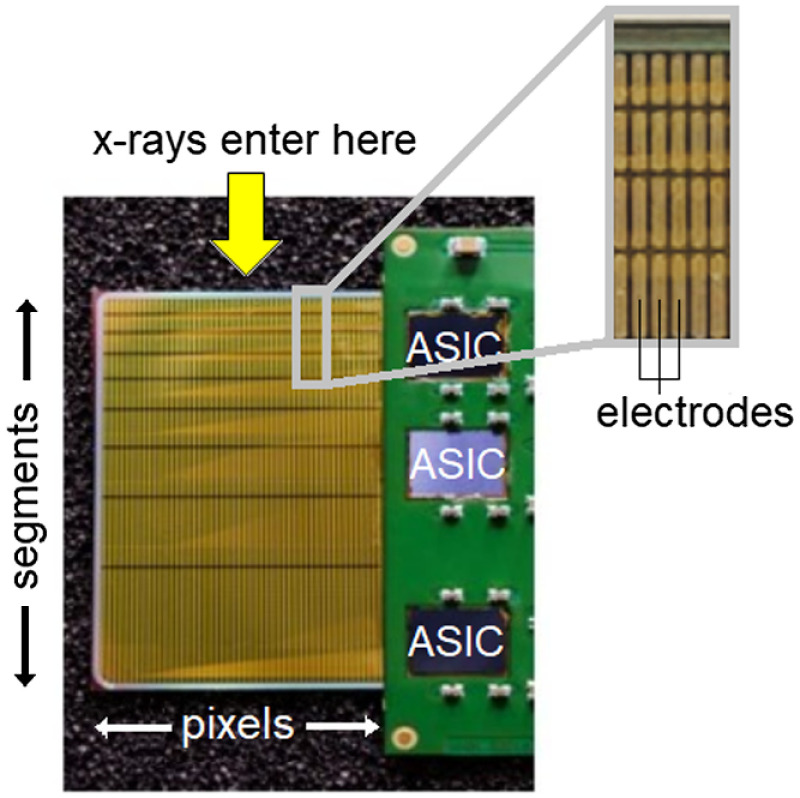
Photograph of a segmented silicon strip detector module. The module is oriented with its upper edge toward the x-ray source. Three wire-bonded ASICs are visible as the black rectangular shapes to the right.

To determine the pulse shape, noise, and gain for each of the two shaping time settings, measurements were performed at Diamond Light Source (United Kingdom), beamlines B16 and I15. These beamlines are able to produce monochromatic x-rays of 4 to 45 keV and 20 to 80 keV, respectively, which make them suitable for detector characterization at diagnostic x-ray energies. The measurement setup included a silicon strip detector with wire-bonded ASICs. Figure 3 shows one such detector prototype with nine depth segments. The detector was mounted in a light-tight box and aligned to the beam in edge-on geometry. The incident x-ray beam was narrowed by collimators to illuminate the center of one pixel, i.e., the center of the projection of the strip as seen from the source, ensuring complete charge collection and eliminating edge effects such as charge-sharing. A bias voltage of 400 V was applied as described by Xu et al.^10^ and measurements were performed on four ASICs in separate detector modules. To keep the noise level approximately equal, only one depth segment in each strip (the one closest to the x-ray source) was used for the measurements.

For each detector module and shaping time setting, the gain was measured by first scanning the lowest digital-to-analog (DAC) threshold voltage across the whole DAC range with monochromatic x-ray beams with a subset of the energies [0, 40, 60, 70, 80] keV where 0 keV represents a noise-only-measurement with no incident x-rays. Modified complementary error functions of the form $f(x,\mu ,\sigma ,A_{1},A_{2},A_{3},A_{4})=\frac{1}{2}\mathrm{erfc}(\frac{x-\mu }{\sqrt{2}\sigma })(A_{1}(x-\mu )+A_{2})+A_{3}(x-\mu )+A_{4}$ were then fitted to the measured data with x as the DAC voltage and $\theta =[\mu \;\sigma \;A_{1}\;A_{2}\;A_{3}\;A_{3}]$ as the fitting parameters. The gain was obtained by fitting a linear relation between the μ values obtained by the fitting and the incident x-ray energies.^10^ The noise level σ in keV was calculated from the fitting to the 0 keV measurement by dividing the obtained value of σ with the calculated gain.

**Fig. 4:**
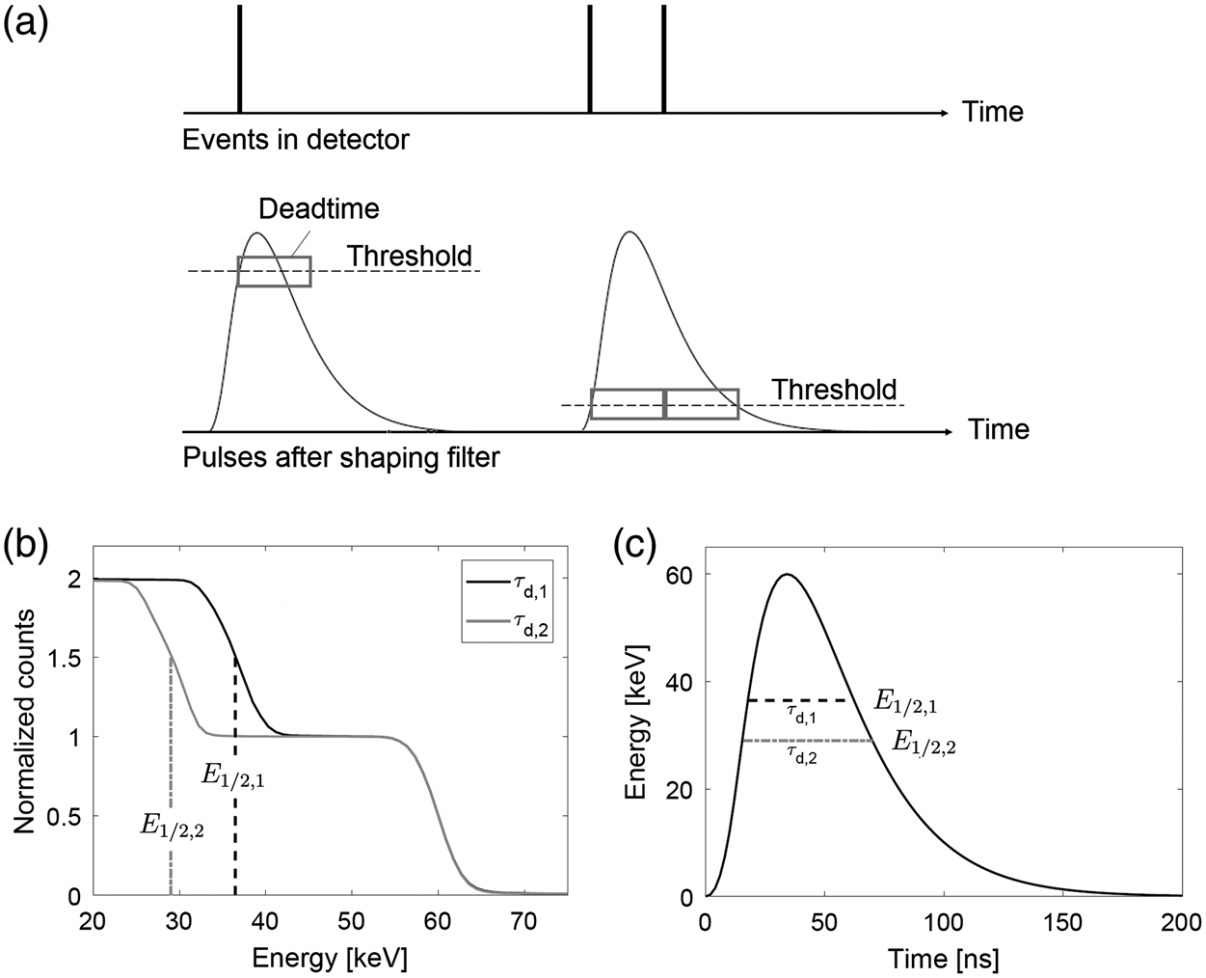
(a) Deadtime behavior of the ASIC. (b) Simulated DAC scans of 60 keV pulses for two different deadtimes $\tau_{d,1}$ and $\tau_{d,2}$. The curves have been normalized according to the number of simulated pulses. Each dashed line shows the threshold energy $E_{1/2}$ where the pulse length is equal to the used deadtime. (c) Simulated 60 keV pulse. The energy coordinate of each dashed line corresponds to the identified energy threshold in the left figure and the pulse length at this energy is equal to the deadtime. (b) and (c) Apply to a case where the ASIC is designed to trigger immediately when the pulse rises above the threshold. For the detector study here, the pulses are sampled with a 10-ns sampling interval, which means that the actual pulse length is on average 5 ns longer than the deadtime used in the measurement.

In the ASIC implementation, there is no direct access to measure the shape of each pulse due to a high sensitivity toward additional load and the bandwidth required to capture the pulses reliably. Therefore, in this paper, the pulse shapes were measured using an indirect method in which the lowest DAC threshold was scanned across the entire DAC range with a monochromatic beam for different deadtime settings. Measurements that are performed with a deadtime that is shorter than the pulse length result in double counting [see Fig. 4(a)]. As the pulse length depends on the threshold energy at which it is measured, a certain deadtime might result in double counts for a certain threshold, while at a higher threshold the pulses are single counted. By scanning the DAC threshold across the entire DAC range, it is possible to observe when pulses transition from being double counted to single counted. The threshold energy at which this occurs, $E_{1/2}$, then represents the energy at which the pulse length is equal to the deadtime. This is depicted in Figs. 4(b) and 4(c) where DAC scans have been simulated for two different deadtimes $\tau_{d,1}$ and $\tau_{d,2}$.

In the limit of an infinitely short clock cycle, the threshold at which the pulse length equals the deadtime, $E_{1/2}$, is found where half of the pulses are counted twice and the rest are not [Fig. 4(b)]. In our ASIC, pulses are sampled at intervals given by the clock cycle $\tau_{c}=10\;\mathrm{ns}$, which means that each count is triggered with a random delay between 0 and $\tau_{c}$ from the moment when the signal crosses over the lowest threshold. The pulse length at $E_{1/2}$ is therefore equal to $\tau_{d}+\tau_{c}/2$, where $\tau_{d}$ is the deadtime.

### 2.3 Simulations

#### 2.3.1 ASIC models

In order to simulate the behavior regarding pulse shapes and noise, we developed a model of our current ASIC prototype. The developed ASIC model is based on the transfer function of the prototype ASIC given by Eq. (2) and Table 1. The ASIC model was simulated in Matlab (The Mathworks inc., Natick, Massachusetts) with s=jω, $C_{f}=200\;\mathrm{fF}$, $C_{\mathrm{pz}}=1600\;\mathrm{fF}$, $C_{s}=200\;\mathrm{fF}$, $\tau_{s}=14\;\mathrm{ns}$, and $I_{\text{in}}=L\{i_{\text{pulse}}(t)\}$ with $i_{\text{pulse}}(t)$ as the input current pulse and L as the Laplace transform.

A second ASIC model of a proposed future ASIC channel was also developed to evaluate the potential performance in terms of power and noise. In the proposed future ASIC model, we use the ideal ASIC representation given by Eq. (2) and Table 1, which corresponds to the same system architecture as our current ASIC prototype assuming a negligible load of the comparators on the filter. This model was evaluated with the same values of $C_{f}$, $C_{\mathrm{pz}}$, and $C_{s}$ as presented above, however, $\tau_{s}$ was set to the same value as $\tau_{0}$ so that any change in shaping time is followed by a corresponding change in the time constant of the shaper amplifier. The proposed future ASIC model was evaluated for different shaping times defined as $\tau =2\tau_{0}$.

The input currents to both ASIC models were created in two steps. Photon interactions and the resulting charge clouds were first simulated using PENELOPE.^19^ A charge transport model including both drift and diffusion was then applied assuming a silicon strip detector with an edge-on geometry as presented by Xu et al.^20^ and a pixel size of $0.4\times 0.5\;{\mathrm{mm}}^{2}$. The charge drift was realized by calculating the drift velocity $v_{d}$ according to $v_{d}=\mu E$ where μ is the charge carrier mobility and E is the electric field. The electric field was obtained by solving the Poisson equation $\nabla \cdot E=-\nabla^{2}\varphi =\frac{\rho }{\epsilon }$ with φ as the potential, ϵ as the dielectric constant of silicon, and ρ as the charge density given by the net doping concentration $4.6\cdot 10^{11}\;{\mathrm{cm}}^{-3}$. The boundary conditions were given by φ=0 at the collecting strip and φ=400  V at the backside of the detector. Charge diffusion was modeled in two dimensions x and y corresponding to the area between the collecting electrodes and the backside of the detector. For each charge carrier, the diffusion distance in x was obtained by sampling from the diffusion equation $P(x)=\frac{1}{\sqrt{4\pi Ddt}}\;\exp(-\frac{x^{2}}{4Ddt})$, with dt as the time step of the charge transport simulation and the diffusion constant $D=\frac{kT\mu }{e}$ with k as the Boltzmann constant, T as the absolute temperature, and e as the fundamental electron charge. Similarly, the diffusion distance in y was obtained by sampling separately from the same distribution. From this, the diffusion velocity $v_{D}$ was calculated as $v_{D}=(dx/dt,dy/dt)$. With the total velocity defined as $v=v_{d}+v_{D}$, the induced currents were calculated in each time step using the Shockley–Ramo theorem.^21^^,^^22^ A time step of 2 ns was chosen throughout all charge transport simulations to limit the required computational power of the ASIC models while maintaining the characteristics of the pulses.

#### 2.3.2 Fitting simulation parameters to measured data

To obtain the correct parameters in the model of the ASIC prototype, pulses were simulated for different values of $\tau_{0}$ and $B_{\text{short}}$ and then compared to the measured data. According to the expected shaping time of 40 ns with the long shaping time setting, we expect $\tau_{0}$ to be 20 ns. However, to account for any discrepancies between the simulation model and the ASICs in the measurements, we assumed $\tau_{0}$ to be in the range between 10 and 30 ns. In order to perform the comparison, pulses were simulated for $\tau_{0}$ and $B_{\text{short}}$ in the ranges of $10\;\mathrm{ns}\leq \tau_{0}\leq 30\;\mathrm{ns}$ in steps of 0.1 ns and $0.5\leq B_{\text{short}}<1$ in steps of 0.01 for each of the monoenergies used in the measurements (40, 60, 70, 80 keV). From each simulated pulse, pulse lengths were extracted at the same energies as for the measured pulse lengths. A mean square error fitting was then performed between the extracted simulated pulse lengths and the measured pulse lengths. Note that the measured pulse height was not used for fitting the pulse shape.

#### 2.3.3 Simulated noise

Both ASIC models were extended to evaluate the effect of the shaping time on the electronic noise. This was performed by including two input noise sources: one in the CSA and one in the filter. The noise sources are typically defined by the thermal noise from the input transistor pair, thermal noise in interconnection resistances, and accumulated thermal noise through the bias chain to the individual amplifiers in the signal chain. The noise in the CSA is also affected by the geometry of the strip and will increase with increasing segment size due to a larger capacitive load at the input. In both ASIC models, the CSA was assumed to be the main noise contributor^17^ and the noise power from each noise source was approximated as 4/5 from the CSA and 1/5 from the filter. With the ASIC output noise power defined as the variance of the output voltage for a zero input current and by assuming no correlation of the noise between the two noise sources, the total output noise can be expressed as

$$\sigma_{\text{total}}^{2}=\sigma_{\mathrm{CSA}}^{2}+\sigma_{\text{filter}}^{2},\;\sigma_{\mathrm{CSA}}=\sqrt{\frac{4}{5}}\sigma_{\text{total}},\;\sigma_{\text{filter}}=\sqrt{\frac{1}{5}}\sigma_{\text{total}},$$ (3)

with $\sigma_{\text{total}}$ as the standard deviation of the total output noise and $\sigma_{\mathrm{CSA}}$ and $\sigma_{\text{filter}}$ as the standard deviation of the output noise from the CSA and the filter, respectively.

In the architecture of both ASIC models, the noise originating in the CSA is unfortunately amplified by the parasitic detector capacitance, but with a slightly different transfer function than the input signal itself. The power-spectral density of the noise at the output of the filter due to the CSA noise current is given by^15^

$${\mathrm{PSD}}_{\text{noise},\mathrm{CSA}}(f)={\left(\frac{1}{G_{f}}\frac{C_{\det}}{C_{f}}\frac{C_{\mathrm{pz}}}{C_{s}}\right)}^{2}\frac{{\left|2\pi f\tau_{s}\right|}^{2}}{{\left|1+2\pi f\tau_{s}\right|}^{2}}\frac{4}{{\left|1+2\pi f\tau_{1}\right|}^{2}}\frac{4}{{\left|1+2\pi f\tau_{2}\right|}^{2}}I_{\text{in},\mathrm{CSA}}^{2}(f),$$ (4)

with $G_{f}=5\;\mathrm{mS}$ as the transconductance of the input transistor of the CSA, $C_{\det}=5\;\mathrm{pF}$ as the detector capacitance, and $I_{\text{in},\mathrm{CSA}}^{2}(f)$ as the input-referred CSA noise current power-spectral density. The time constant of the shaper amplifier, $\tau_{s}$, and the time constants of the two $G_{m}\text{-}C$ stages, $\tau_{1}$ and $\tau_{2}$, are given by Table 1 for each ASIC model. Correspondingly, the filter noise is modeled as an input-referred noise source at the filter input, which results in an expression corresponding to the filter part of Eq. (2):

$${\mathrm{PSD}}_{\text{noise},\text{filter}}(f)=\frac{4}{{\left|1+2\pi f\tau_{1}\right|}^{2}}\frac{4}{{\left|1+2\pi f\tau_{2}\right|}^{2}}V_{\text{in},\text{filter}}^{2}(f),$$ (5)

with ${\mathrm{PSD}}_{\text{noise},\text{filter}}(f)$ as the filter output noise power spectral density and $V_{\text{in},\text{filter}}^{2}(f)$ as the filter input noise spectral density. The total output noise is given by ${\mathrm{PSD}}_{\text{noise}}(f)={\mathrm{PSD}}_{\text{noise},\mathrm{CSA}}(f)+{\mathrm{PSD}}_{\text{noise},\text{filter}}(f)$.

The input noise from each noise source (CSA and filter) was realized in the time domain as Gaussian white noise with zero mean and a standard deviation of 0.45  μA rms and 2.72 mV rms for the CSA and filter, respectively. In the case of the proposed future ASIC model, this corresponds to a total output noise level of σ=2  keV with a shaping time of 40 ns. The noise from each noise source was Fourier transformed by applying the fft in Matlab and then propagated to the output by the channel’s transfer function from the CSA and filter inputs, respectively. The noise output power was then obtained by integrating the resulting noise power spectral densities over frequency. The time-domain output noise was obtained by applying the discrete inverse Fourier transform ifft to the propagated output signal.

#### 2.3.4 Dose efficiency

In this work, dose efficiency was taken to be proportional to the registered fraction of incident photons, thereby giving the dose efficiency for a purely photon-counting detector without including energy information. Dose efficiency was first considered in the absence of pileup by evaluating the effect of the lowest threshold on the number of registered counts. This was performed by simulating spectrums of the deposited energies in a silicon detector for three different thicknesses of soft tissue filtration between the x-ray source and detector: 20, 30, and 40 cm. Each spectrum was simulated by combining the spectrum from an x-ray tube with the simulated energy response of a silicon detector. The x-ray tube spectrum was modeled based on SRS-78^23^ with a tube voltage of 120 kVp, a 10.5-deg tungsten anode, 8.38 mm aluminum filtration, 0.8 mm beryllium, along with the soft tissue filtration. The silicon detector energy response was simulated in GATE.^24^ The detector was modeled as a silicon strip detector with an edge-on geometry such as the detector presented by Liu et al.^25^ A pixel size of $0.4\times 0.5\;{\mathrm{mm}}^{2}$ was used with a strip length of 30 mm segmented into nine depth segments, including a 0.6-mm dead layer at the pixel top. Based on the resulting spectrum shapes, the dose efficiency was evaluated at different thresholds by calculating the ratio between the number of counts above the threshold and the total number of counts within the entire spectrum. This dose efficiency represents the dose efficiency obtained at a low incident x-ray flux where all pulses are well separated in time and there is no pileup. To relate the lowest threshold to noise, the lowest threshold was set to 4σ, which corresponds to one noise count on average per frame time of 150  μs.

The proposed future ASIC model was then used to evaluate the count characteristics for different shaping times. This was performed by simulating the output count rate for different input count rates. The input current was modeled as pulse trains based on pulses from PENELOPE and the described charge transport model. The photon energies used in the PENELOPE simulations were sampled from the combined spectrum of an x-ray tube and the simulated energy response of a silicon detector with the x-ray tube operated at 120 kVp and 20 cm soft tissue filtration between the x-ray source and the detector. Each pulse train was discretized in 2 ns steps and the total frame time of each pulse train was set to T=150  μs. In every pulse train, the number of interacting photons, N, was drawn from a Poisson distribution with mean fT, where f is the photon flux. The time stamp of each photon interaction was sampled according to a uniform distribution on the interval [0:T]. Shaping times of 40, 100, and 200 ns were used and counts were registered during a total time of 1000 frames. As the shaping time affects both the pulse length and the noise level, the deadtime was chosen in order to avoid double-counting the pulse tails at the lowest threshold. This was performed by assuming a lowest threshold of 4σ and setting the deadtime to the length of a 100 keV pulse. For each shaping time, σ was determined based on the same input noise source levels (see Sec. 2.3.3.).

#### 2.3.5 Power consumption

The effects of power consumption and shaping time on the noise level were studied in order to evaluate the potential in using the shaping time to counteract the noise resulting from a decreased power consumption in the preamplifier.

The relation between power consumption and noise in the preamplifier was modeled using an approximation^26^

$$\sigma \propto \frac{1}{\sqrt{P}},$$ (6)

where σ is the standard deviation of the output noise and P is the power to the preamplifier.

Noise as a function of shaping time was simulated using the proposed future ASIC model. For this, a zero input current was used along with the reference noise value of σ=2  keV for a shaping time of 40 ns. For each shaping time, the noise level σ was calculated as the standard deviation of the time-domain output noise.

## 3 Results

### 3.1 Measurements

The measured gain in mV/keV is presented in Table 2 for each shaping time setting and detector module. The table also shows the standard deviation σ of the noise. These values were obtained by fitting modified complementary error functions to the measured data.

**Table 2:** Measured gain and noise for each shaping time setting and detector module.

Detector module	Gain for $\tau_{\text{long}}$ (mV/keV)	Gain for $\tau_{\text{short}}$ (mV/keV)	$\sigma_{\text{long}}$ (mV)	$\sigma_{\text{short}}$ (mV)
1	1.45	1.89	3.99	5.50
2	1.54	2.00	3.48	4.92
3	1.51	1.89	3.40	4.58
4	1.63	2.10	3.73	5.01

**Fig. 5:**
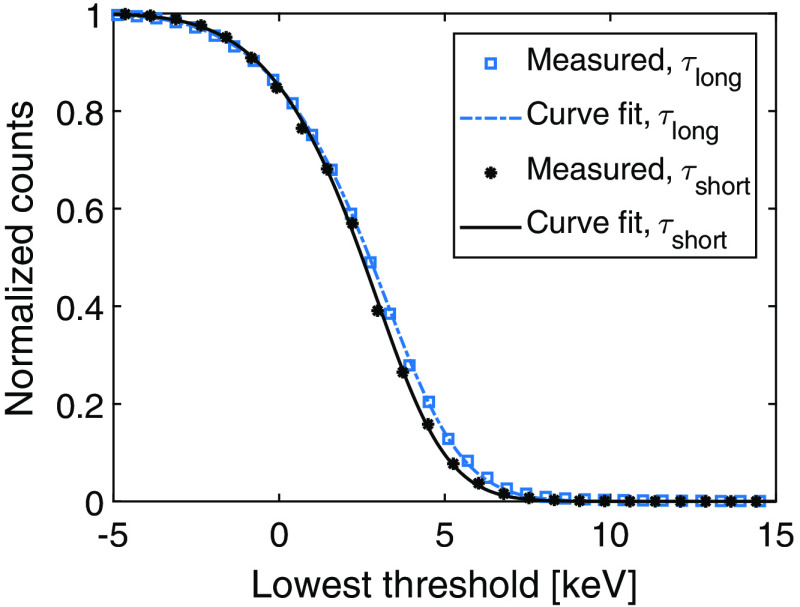
Measured noise in one detector module using different shaping time settings along with fitted modified complementary error functions $\frac{1}{2}\mathrm{erfc}(\frac{x-\mu }{\sqrt{2}\sigma })(A_{1}(x-\mu )+A_{2})+A_{3}(x-\mu )+A_{4}$ with x as the lowest threshold in keV.

To enable analysis and comparison between the two shaping time settings, the determined gain was used to convert the measured noise from mV to keV. The calibrated noise values for each detector module and shaping time are presented in Table 3. In Fig. 5, the measured noise curves from one detector module are presented along with fitted modified complementary error functions.

**Table 3:** Measured noise in keV for the long and short shaping time settings.

Detector module	$\sigma_{\text{long},\mathrm{keV}}$ (keV)	$\sigma_{\text{short},\mathrm{keV}}$ (keV)
1	2.76	2.90
2	2.26	2.46
3	2.25	2.43
4	2.29	2.38

**Fig. 6:**
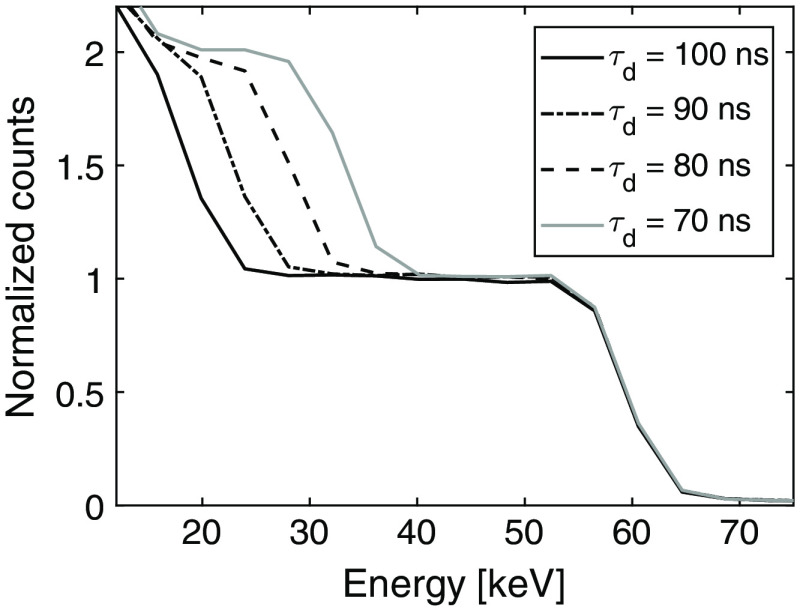
Threshold scan to obtain the pulse shape at 60 keV using four different deadtimes and the long shaping time setting.

Threshold scans to obtain the pulse shape were performed for the energies and deadtimes presented in Table 4. In Fig. 6, the data from one such threshold scan with the long shaping time and a beam energy of 60 keV are shown. The curves have been normalized by the number of unique counts, measured as the height of the plateau between 40 and 55 keV in Fig. 6. Analogously to the calibrated noise, all threshold scans have been converted from mV to keV using Table 2.

**Table 4:** Energy and deadtime sets used in the threshold scans to obtain the pulse shapes.

	$\tau_{\text{long}}$		$\tau_{\text{short}}$	
Detector module	Measured energies (keV)	Used deadtimes (ns)	Measured energies (keV)	Used deadtimes (ns)
1	40, 60, 70	70, 80, 90^a^, 100^a^	60, 70	70^a^, 80^a^
2	40, 60, 80	70, 80, 90, 100^a^	40, 60	70, 80, 90^a^
3	40	40, 50, 60, 70, 80, 90, 100	40	40, 50, 60, 70
4	40	40, 50, 60, 70, 80, 90, 100	40	40, 50, 60, 70

a Deadtimes used only for energies 60, 70, and 80 keV.

**Fig. 7:**
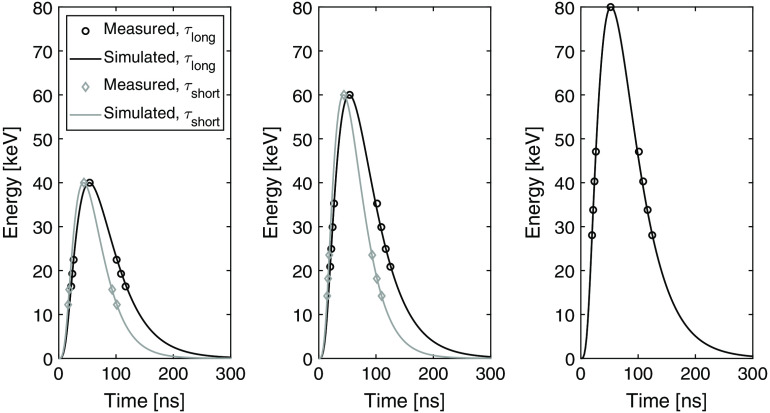
Measured pulse lengths with different shaping time settings for one detector module along with simulated pulse shapes. The parameters of the ASIC model used to simulate the pulse shapes have been fitted such that the pulse amplitudes agree with the nominal energies and the pulse shapes agree with the measured pulse lengths.

The resulting simulated pulse shapes for one detector module are shown in Fig. 7 together with measured data points for the positive and negative flank of the pulse. The energy coordinate of each pair of data points corresponds to the threshold energy and the horizontal distance between the points in the pair is the used deadtime. The fitted shaping times are presented in Table 5.

**Table 5:** Determined shaping times from the curve fit to the measured pulse lengths.

Detector module	$\tau_{\text{long}}$ (ns)	$\tau_{\text{short}}$ (ns)
1	39.2	27.8
2	41.4	28.6
3	38.4	27.6
4	38.6	28.2

In Table 6, the measured noise ratios and gain ratios between the long and the short shaping times are presented along with the corresponding simulated values. The noise ratio for each detector module, $\sigma_{\text{long},\mathrm{keV}}/\sigma_{\text{short},\mathrm{keV}}$, is the ratio between the values in Table 3 and the gain ratio, G, is the ratio between the gain values for the long and the short shaping time settings presented in Table 2. The corresponding simulated values were obtained from the simulated ASIC model using the determined shaping times in Table 5.

**Table 6:** Measured noise and gain ratios, G, for the two shaping times along with the corresponding simulated values.

Detector module	$\sigma_{\text{long},\mathrm{keV}}/\sigma_{\text{short},\mathrm{keV}}$	Simulated $\sigma_{\text{long},\mathrm{keV}}/\sigma_{\text{short},\mathrm{keV}}$	G	$G_{\text{Simulated}}$
1	0.95	0.95	0.76	0.77
2	0.92	0.94	0.77	0.75
3	0.92	0.95	0.80	0.78
4	0.96	0.95	0.78	0.79

### 3.2 Simulated Performance of Proposed Future System

#### 3.2.1 Dose efficiency

**Fig. 8:**
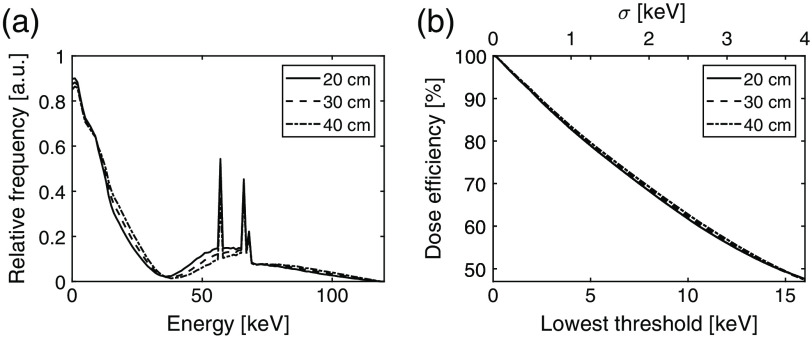
(a) Simulated spectrums of the deposited energies in a silicon detector from an x-ray source operated at 120 kVp including 20, 30, and 40 cm soft tissue filtration between the source and the detector. (b) Dose efficiency as a function of lowest threshold for the three spectrum shapes normalized to 100% at a 0 keV threshold. The dose efficiency is the fraction of interactions registered above the lowest threshold.

The dose efficiency based on the lowest threshold was calculated for the three spectrums in Fig. 8(a), which shows the deposited energies in a silicon detector with 20, 30, and 40 cm soft tissue filtration between the x-ray source and the detector. The dose efficiency as a function of the lowest threshold is shown in Fig. 8(b). Assuming a lowest threshold of 4σ, the mapping from lowest threshold to σ is displayed as an additional axis in the figure.

**Fig. 9:**
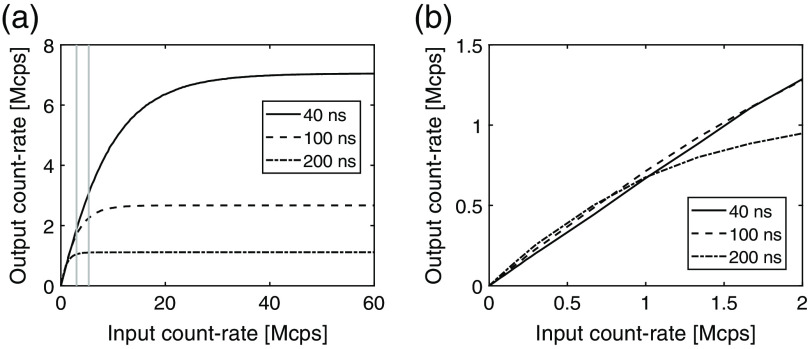
Count characteristics for different shaping time values on different input count rate intervals. The vertical lines show (from left to right) the input count rate for a detector with 16 and 9 segments, respectively, assuming an incident count rate of $300\;\mathrm{Mcps}/{\mathrm{mm}}^{2}$ and a detection efficiency of 80%.^8^^,^^10^ The spectrum of input pulses was obtained from a 120-kVp x-ray tube spectrum assuming 20 cm soft tissue filtration together with the response function of the silicon detector.

In order to evaluate the dose efficiency related to high count rates, the lowest threshold and deadtime were determined for each shaping time and are presented in Table 7 along with the dose efficiency resulting from the lowest threshold. The count characteristics are shown in Fig. 9 and were obtained by simulating the registered number of counts at different input count rates for each shaping time with the corresponding threshold and deadtime.

**Table 7:** Lowest threshold and deadtime values for each shaping time. The lowest threshold was set in relation to the noise level σ as 4σ. The deadtime corresponds to the length of a 100-keV pulse at the lowest threshold. The dose efficiency is the fraction of interactions registered above the lowest threshold.

Shaping time (ns)	Lowest threshold (keV)	Deadtime (ns)	Dose efficiency (%)
40	8	140	68.8
100	5.2	370	78.5
400	1.9	890	92.1

#### 3.2.2 Power consumption

**Fig. 10:**
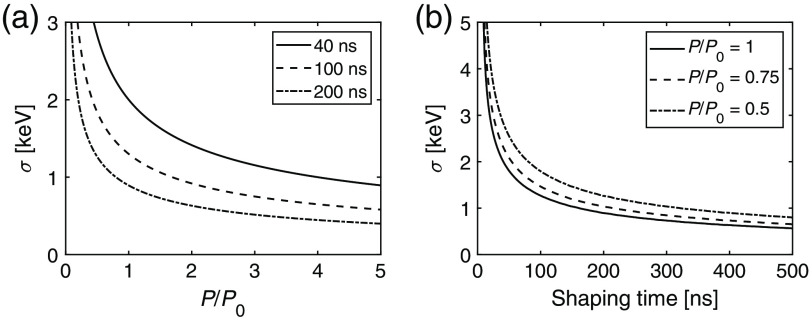
(a) Noise as a function of power for different fixed shaping times. (b) Noise as a function of shaping time for different fixed power levels.

**Fig. 11:**
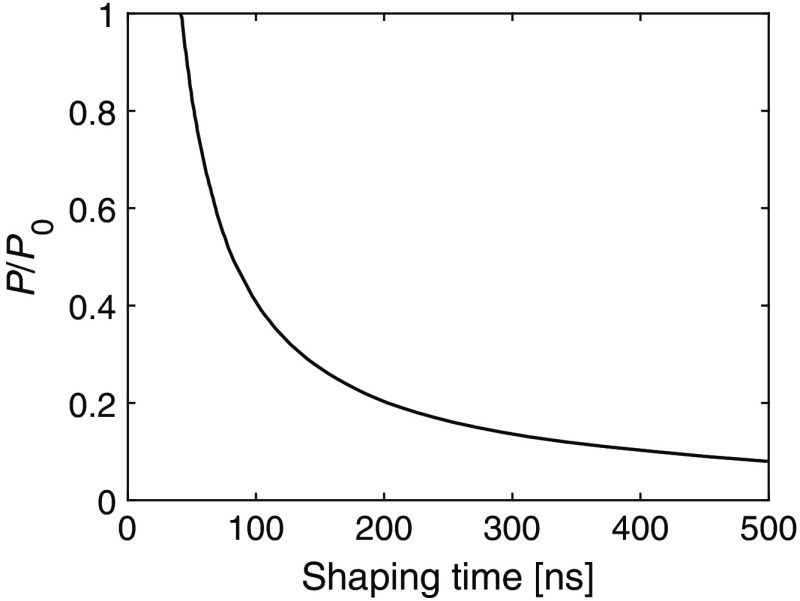
Power consumption and required shaping time to maintain a constant noise level of σ=2  keV.

Noise as a function of power is presented in Fig. 10(a) for the different shaping time values. The power values are given as multiples of $P_{0}$, which is the power level that gives a noise level of σ=2  keV at a 40-ns shaping time. In Fig. 10(b), the noise levels for three different power settings are shown as functions of shaping time. To show the potential for decreasing the power consumption by increasing the shaping time while keeping the noise constant, the resulting relation between power consumption and shaping time is shown in Fig. 11.

## 4 Discussion

As seen in Table 2, the gain is lower for the long shaping time than for the short shaping time. This is expected as the low-pass filtration decreases the pulse height. The measured noise values in Table 2 also show a reduction with the long shaping time. The small variations that can be seen between the measured gain and noise values for different detector modules could be caused by fabrication imperfections in the ASICs or statistical errors in the measurements. Based on the calibrated noise presented in Table 3 and Fig. 5, the long shaping time results in an average noise decrease of 6.1% compared to the short shaping time. This corresponds to an SNR increase of 6.5%, and it is clear that even though the gain decreases when the shaping time is increased, the noise decreases more, and the resulting SNR becomes higher.

The measured counts as a function of the threshold for different deadtime settings in Fig. 6 are similar to the simulated curves in Fig. 4. However, at ∼15  keV, the normalized counts become larger than 2 resulting from triple counted pulses and noise counts. These data points have been excluded in the curve fitting.

The two $G_{m}\text{-}C$ stages that form the filter in our ASIC are expected to give a semi-Gaussian pulse shape as described by the system transfer function [Eq. (2)] used in the simulation model. The measured pulse lengths in Fig. 7 show good correspondence with this predicted pulse shape and similar fitting results were obtained for all ASICs. Note that as the pulse shape was measured by measuring the pulse length at different pulse heights, the relative time points of the measurements for different deadtime settings are not known, i.e., there is not sufficient information to determine if the pulse rises faster than it drops off. However, the measurements are consistent with the theoretical model.

The determined shaping times (Table 5) result in an average shaping time of 39.4 ns with the long shaping time setting and 28.1 ns with the short shaping time setting. When the determined shaping times are used to simulate the noise ratio, $\sigma_{\text{long}}/\sigma_{\text{short}}$, and the gain ratio, G, the resulting values agree well with the measured values as presented in Table 6. The discrepancy could be caused by small differences in the performed curve fittings due to statistical errors in the measurements. Overall, the ASIC model is able to describe the measurements well, and this indicates the potential of using the model in future investigations.

The deadtimes in this work were chosen in order to obtain double counts at each beam energy. The number of deadtimes was determined in relation to the available synchrotron beam time during each synchrotron session. To improve the capture of the pulse shape, the number of deadtimes could be increased as this would provide information about the pulse length at more energy levels. However, as the deadtime is a multiple of the clock cycle and the resulting double counts must arise in the threshold range between the noise floor and the energy of the pulse, the number of possible deadtimes is limited. This could be mitigated by utilizing a shorter clock cycle, but this would put much harder constraints on, e.g., the speed of the digital readout circuitry as well as the bandwidth of the analog circuitry.

The dose efficiency based on the lowest threshold presented in Fig. 8 shows that the dose efficiency is largely independent of the soft tissue thickness. It is also clear that there is a benefit in dose efficiency if the lowest threshold is decreased. Reducing the threshold from, e.g., 8 to 4 keV increases the dose efficiency from 68.8% to 85.3%.

**Fig. 12:**
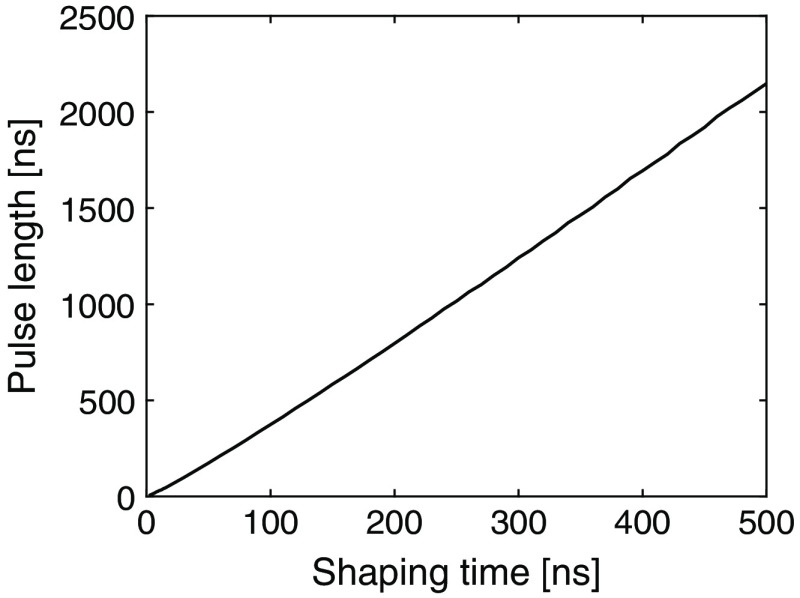
Pulse length of a 100-keV pulse at a threshold of 4σ for different shaping times.

The dose efficiency at high input count rates depends on the pulse length and the corresponding deadtime. In Fig. 12, the relation between shaping time and pulse length is presented. As increasing the shaping time results in longer pulses and thereby a longer deadtime, the count characteristics in Fig. 9(a) show that the short shaping time has the best count-rate performance at high input count rates. Saturation occurs when the curves become flat and represents the maximum count-rate capability of the channel. Due to the short pulse length, the 40-ns shaping time saturates at a higher input count rate. However, at low input count rates, the lower threshold levels enabled by the 100- and 200-ns shaping times provide better performance and give higher output count rates compared to the 40-ns shaping time. This can be seen in Fig. 9(b).

In Fig. 10(a), noise is described as a function of power for different shaping times. From the figure, it is clear that the noise level decreases as the input power increases. Correspondingly, Fig. 10(b) shows how the noise level decreases when the shaping time is increased. The resulting relation between power consumption and noise in Fig. 11 shows that it is possible to counteract the additional noise caused by decreasing the power consumption by simultaneously increasing the shaping time. The potential for decreasing the noise by increasing the shaping time is particularly high if the initial shaping time is low. If the starting point is 200 ns, a 50% decrease in power consumption can be counteracted by increasing the shaping time by a factor of 2.5 to 500 ns. At a starting point of 40 ns, the corresponding required shaping time increase is a factor of 1.875 to 75 ns.

For future work, there are a number of ways in which varying the shaping time could be used to increase the dose efficiency. One example is to adjust the shaping time for each projection line based on the flux. The pulse length would then be adapted to the count-rate demand. Another example is a dual or multiple shaper system in which two or more filters could be implemented in parallel to process the same signal. This could be, e.g., one filter of a short shaping time to enable a high count-rate capability and one filter of a longer shaping time to measure low-energy interactions. Such an implementation would increase the dose efficiency while maintaining a high count-rate capability for all projection lines.

## 5 Conclusions

We have presented noise and pulse shape measurements for a photon-counting ASIC with two different shaping times. Our results show that increasing the shaping time from 28.1 to 39.4 ns decreases the noise and increases the SNR with 6.5% at low count rates. At the same time, the pulse length increases, which results in a lower count-rate capability. This shows that adjusting the shaping time can be an important tool to adapt the pulse length and noise level to the photon flux and thereby optimize the dose efficiency of photon-counting silicon detectors. With our proposed future ASIC design, we also show that it is possible to maintain a constant noise level while decreasing the power consumption by 50% by increasing the shaping time with a factor of 1.875.
